# Heme Degradation by Heme Oxygenase Protects Mitochondria but Induces ER Stress via Formed Bilirubin

**DOI:** 10.3390/biom5020679

**Published:** 2015-04-30

**Authors:** Andrea Müllebner, Rudolf Moldzio, Heinz Redl, Andrey V. Kozlov, J. Catharina Duvigneau

**Affiliations:** 1 Institute for Medical Biochemistry, Veterinary University Vienna, Veterinaerplatz 1, 1210 Vienna, Austria; E-Mails: andrea.muellebner@vetmeduni.ac.at (A.M.); rudolf.moldzio@vetmeduni.ac.at (R.M.); 2 Ludwig Boltzmann Institute for Experimental and Clinical Traumatology, Donaueschingenstraße 13, 1200 Vienna, Austria; E-Mails: office@trauma.lbg.ac.at (H.R.); andrey.kozlov@trauma.lbg.ac.at (A.V.K.)

**Keywords:** heme oxygenase activity, bilirubin, hemin, endoplasmic reticulum, mitochondria

## Abstract

Heme oxygenase (HO), in conjunction with biliverdin reductase, degrades heme to carbon monoxide, ferrous iron and bilirubin (BR); the latter is a potent antioxidant. The induced isoform HO-1 has evoked intense research interest, especially because it manifests anti-inflammatory and anti-apoptotic effects relieving acute cell stress. The mechanisms by which HO mediates the described effects are not completely clear. However, the degradation of heme, a strong pro-oxidant, and the generation of BR are considered to play key roles. The aim of this study was to determine the effects of BR on vital functions of hepatocytes focusing on mitochondria and the endoplasmic reticulum (ER). The affinity of BR to proteins is a known challenge for its exact quantification. We consider two major consequences of this affinity, namely possible analytical errors in the determination of HO activity, and biological effects of BR due to direct interaction with protein function. In order to overcome analytical bias we applied a polynomial correction accounting for the loss of BR due to its adsorption to proteins. To identify potential intracellular targets of BR we used an *in vitro* approach involving hepatocytes and isolated mitochondria. After verification that the hepatocytes possess HO activity at a similar level as liver tissue by using our improved post-extraction spectroscopic assay, we elucidated the effects of increased HO activity and the formed BR on mitochondrial function and the ER stress response. Our data show that BR may compromise cellular metabolism and proliferation via induction of ER stress. ER and mitochondria respond differently to elevated levels of BR and HO-activity. Mitochondria are susceptible to hemin, but active HO protects them against hemin-induced toxicity. BR at slightly elevated levels induces a stress response at the ER, resulting in a decreased proliferative and metabolic activity of hepatocytes. However, the proteins that are targeted by BR still have to be identified.

## 1. Introduction

Heme oxygenase (HO), residing at the endoplasmic reticulum membrane, is the rate-limiting enzyme in the degradation of heme, yielding equivalent amounts of carbon monoxide (CO), ferrous iron (Fe^2+^), and biliverdin (BV). BV is subsequently reduced to bilirubin (BR) by the cytosolic BV reductase (BVR). Stressful conditions lead to an increase in HO activity due to induction of HO-1 [1], a member of the heat shock protein family (HSP32). Up-regulation of HO in the liver is caused by multiple stimuli that include cytokines, bacterial toxins, hypoxia, and increased amounts of the HO substrate, protoheme IX (heme). HO-1 was shown to mediate tissue protection, since its inhibition increased tissue injury, while tissues were protected when HO-1 was upregulated prior to an acute experimental insult [2]. The cytoprotective effects of HO-1 are partly attributed to the degradation of excessive free heme and partly to the generation of the heme degradation products CO and BR, which are able to mimic HO-1-mediated effects [3,4,5]. Although BR was found to exert anti-oxidant activity, which together with BV effectively protects the water/membrane interface [6,7], it is not clear to what extent BR formed in the HO reaction may contribute to the protection against heme-induced oxidative damage to subcellular structures.

Besides its relevance as diagnostic marker for liver diseases [8], BR was for a long time considered a waste product of heme degradation. However, elevated levels of unconjugated BR are able to induce cytotoxic effects, which are well documented for developing neuronal cells [9,10,11,12]. Free unconjugated BR was found to unbalance the redox homeostasis [13], or to affect the mitochondrial membrane integrity and to induce apoptosis [14]. The liver is one of the organs with a relatively high HO activity and involved in the elimination of BR. Thus, especially under conditions of elevated HO activity BR levels may exceed physiologic levels.

Determination of HO activity by means of BR quantification following the classical photometric extraction assay [15,16,17] is sensitive to higher protein concentrations making direct comparison between different sample types difficult. Thus, improvements of these assays should take the high affinity of lipophilic BR to proteins into account. Additionally, this affinity suggests that functional interaction with lipid and protein-rich structures, such as mitochondria or ER, are likely to occur. Increased levels of BR are formed during enhanced HO activity [5] and may target intracellular structures. However, it is not clear whether such an interaction would contribute to protective effects of the HO reaction or whether it may compromise cellular function and thereby limit the cytoprotective properties of the HO reaction. In order to approach this topic we addressed the following questions using rat liver, cultured hepatocytes and isolated mitochondria as *in vitro* systems:

(1) How to account for the amount of BR that is adsorbed by protein and thus not considered when applying the classical photometric extraction assay for the determination of HO activity?
(2) Are the *in vitro* model systems suitable to investigate the effects of BR that is released following HO reaction?
(3) Does the HO reaction rescue hepatic mitochondria from hemin-mediated toxicity?
(4) Is the anti-oxidative property of BR involved in the protective effect of HO towards mitochondria?
(5) How does BR formation relate to the metabolic activity and the proliferative response of cultured hepatocytes under conditions of accelerated HO activity?
(6) Which subcellular structure in the hepatocyte is sensitive to increased levels of BR?

## 2. Aims of This Study

This study aimed at determining the potential limits of the protective range of the HO reaction in liver cells due to the formation of BR. In contrast to previous reports we focus this study more on the biological/analytical impact of the high affinity of BR to proteins in the liver. We consider two major consequences of the high affinity, namely errors in the determined quantity of BR as a measure for HO activity and the direct interaction of BR with mitochondria and ER. These effects were investigated using rat livers, cultured hepatocytes, and isolated mitochondria.

## 3. Results and Discussion

At increased protein concentrations the precise quantification of BR is a problem, because BR may adsorb to proteins, as known for albumin [18]. In the first part of the study we focused on the improvement of the HO assay, since determination of HO activity using BR quantification was compromised by higher protein concentrations [19].

### 3.1. Protein Adsorption of BR and Subsequent Interference with the Quantification Can Be Corrected Using a Polynomial that Accounts for the Protein Amount Present in the Assay

Quantification of BR, the end product from the HO/BVR reaction, is least laborious and therefore the most frequently used approach to determine HO activity [15,16]. BV and its reduction product BR are components exclusively formed by the HO/BVR system and generally tissue or cell homogenates possess sufficient BVR activity assuring the complete conversion of BV to BR and thus allowing the determination of BR by means of HPLC [20] or by photo spectroscopy [15,17].

Although extraction of BR from the aqueous phase into an organic solvent [17] significantly enhances the sensitivity of the assay, because BR is the only component absorbing around 450 nm in the organic extract, the load of unspecific protein has to be reduced, as it was shown to interfere with the assay [19]. Therefore generally microsomal-enriched fractions are prepared, which contain less protein [21]. However, the use of microsomal preparations bears the risk of partially losing HO activity. It was shown that pathogenic stimuli may induce translocation of HO-1 into the cell nucleus [22] or into mitochondria [23]. This translocation increased the enzymatic activity to convert heme in the target compartment [23], while the activity in the microsomal fraction decreased [22].

The problem to correctly quantify BR can be solved in two ways. Either BR calibration curves are used, which contain the same amount of protein, as was the case in a recently presented study for the determination of BR by ELISA [24], or the effect of protein adsorption has to be considered using a mathematical approach. The latter has the advantage of circumventing the laborious and time-consuming preparation of appropriate calibration curves. However, both approaches allow an improved comparison of the capacities of cells or tissues to convert heme, since preparation steps that may introduce biases are reduced.

**Figure 1:**
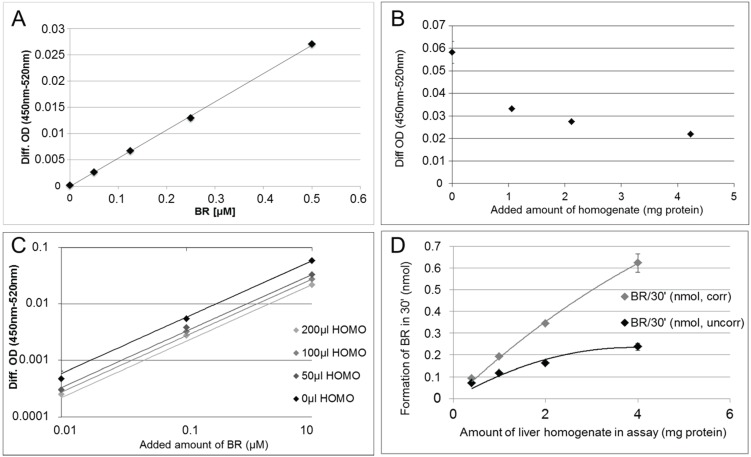
The tight interaction of BR with protein leads to underestimation of enzyme activities using the classical HO-assay. (**A**) Relationship between input and extractable amount of BR from HO-assay buffer (amount of BR was calculated using OD at 450 nm corrected for background OD at 520 nm (Diff. OD)) was linear (no protein added); (**B**) Presence of protein (liver homogenate: HOMO) in assay buffer supplemented with BR (1 µM) decreased the extractable amount of BR (Diff. OD); (**C**) Relationship between input and extractable amount of BR (0.01–1 µM) from HO-assay buffer (Diff. OD) was linear at constant protein concentration (added tissue homogenate (HOMO) with constant protein concentration of 10 mg protein/mL); (**D**) Using the polynomial for correcting the BR amount, the activity of HO (formation of BR/30 min) depended nearly linearly on the amount of liver homogenate used for the assay.

To quantify BR formed by the HO reaction we used calibration curves which we obtained by adding known amounts of BR to an equivalent amount of assay buffer followed by extraction into chloroform (Figure 1A). When adding protein the amount of BR extractable from the buffer decreased in a non-linear fashion (Figure 1B). At constant protein concentrations, however, the relation between input BR and extractable BR remained linear (Figure 1C). Therefore it was possible to develop a polynomial for calculating a correction factor f, which takes into account the adsorption of BR to protein, which is dependent on the amount x of protein.

The corrected BR amount is: br_corr_ = br × f
br = BR concentration (calculated from the calibration curve using the differential OD)
f = −0.076 × x^2^ + 0.704 × x + 1.027
x = protein content present in the assay in mg

Using this equation, we were able to achieve a nearly linear relationship between the amount of BR formed in the reaction and the amount of tissue homogenate subjected to the assay for determination of HO-activity (Figure 1D). The data presented in Figure 1 show that the high affinity of BR to proteins may result in underestimation of HO-activity, which can be corrected using the polynomial. In addition to improvement of the analytical procedure determining HO activity, this result stimulated us to explore the biological impact of a presumed interaction of BR with intracellular protein. Since BR is formed by the HO/BVR reaction within the cell, in close vicinity to the ER, we focused our studies on the effects of BR on mitochondria and ER, structures that are rich in protein and membrane lipids.

### 3.2. BRL3A Cells Have Similar HO Activities as Liver Tissue

**Figure 2:**
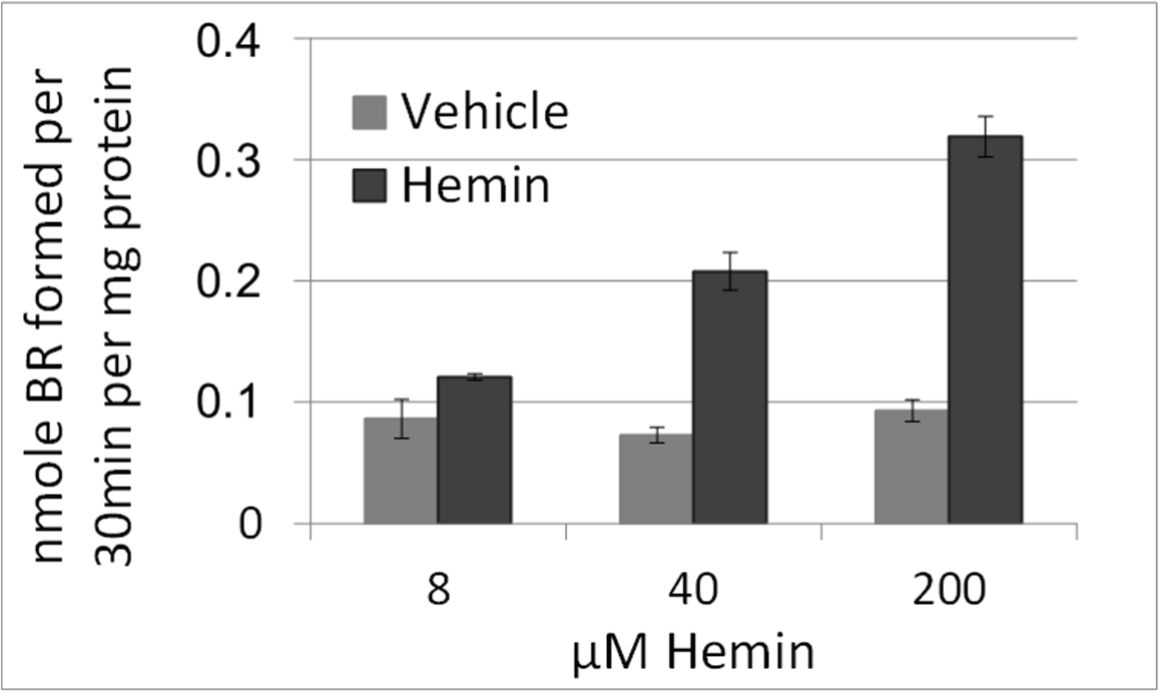
HO activity in hepatocytes after treatment with hemin. Cells were treated with vehicle (DMSO, grey bars) or hemin (8, 40, 200 µM, black bars) for 16 h. HO activity was determined in homogenized cells as described in the Materials and Methods section. The capacity to convert hemin increased in function to the concentration present in medium. HO activity, expressed as capacity of 1 mg cell protein to produce BR was similar to that obtained in the liver of control rats (*n* = 5, dashed line). Data are given as means (±SD) obtained from one experiment with *n* = 2 replicates.

We first verified the suitability of the hepatocyte line BRL3A regarding its HO activity, since we aimed at studying the effects of BR formed by the HO reaction in a cell culture model. In the liver different cell types contribute to the activity of HO, composed of the activity of both enzymes, HO-1 and HO-2. Under physiological conditions the determined HO activity nearly exclusively consists of the activity of HO-2, while an increase accounts for the induction of HO-1, which occurs in all liver cells to different degrees [25]. Since the amount of BR formed depends on the level of the HO activity, we first examined whether BRL3A cells would be able to convert heme at comparable rates as homogenized liver. Cells were cultured and treated with various amounts of hemin, that is protoporphyrin IX containing ferric iron, or vehicle for 16 h, and examined for HO activity as described in the Materials and Methods section (Figure 2). Basal levels of HO activity (Figure 2, grey bars) were similar to those found in homogenates obtained from livers of control rats (dashed line in Figure 2). An incubation for 16 h with varying concentrations of hemin resulted in a dose dependent increase in HO activity, indicative for HO-1 induction (Figure 2, black bars).

These findings show that BRL3A cells are suitable to study the role of HO and the effects mediated by the products of heme degradation.

### 3.3. HO Reaction Rescues Mitochondria from Hemin-Mediated Impairment of Respiration and Subsequent Fragmentation

We next questioned whether HO in BRL3A cells would protect mitochondria of BRL3A cells against hemin-induced toxicity via formed BR. It is known that mitochondria are particularly sensitive to increased intracellular levels of heme [26]. Although heme may reach much higher concentrations, we used levels that have been reported previously as the intracellular threshold for inducing heme toxicity [26].

**Figure 3:**
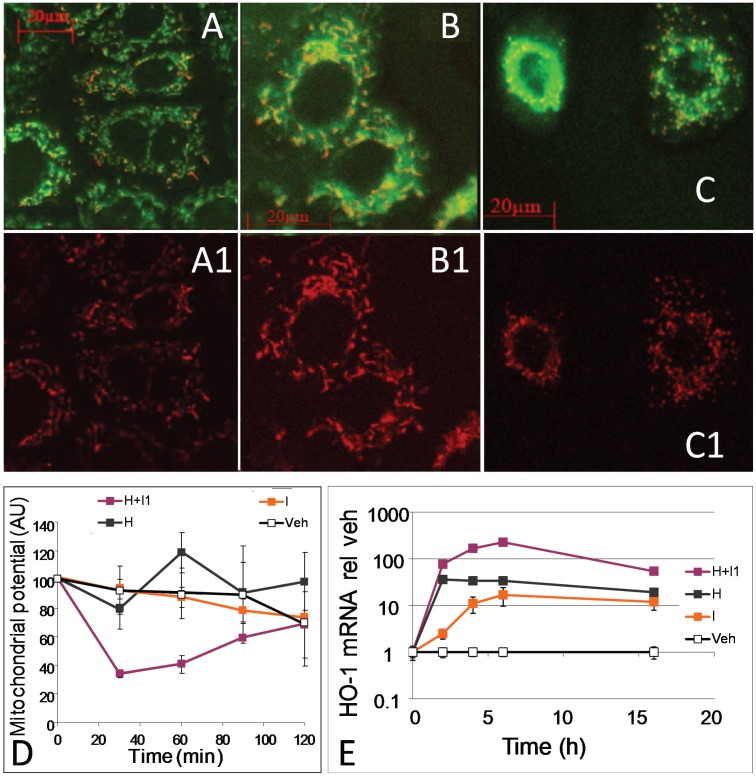
Effect of hemin and zinc protoprophyrin IX on mitochondrial function and morphology in hepatocytes. Liver cells (BRL3A) were incubated with JC-1 for 30 min, thereafter vehicle (DMSO, **A**) or hemin (20 µM); (**B**,**C**)) was added. Zinc protoporphyrin (ZnIXPP), a competitive inhibitor of HO (0.2 µM); was added 10 min before (**C**). Cells were analyzed after 2 h. **A1**, **B1** and **C1** show energized mitochondria (fluorescence at 590 nm). Competitive inhibition of HO by ZnIXPP resulted in delayed fragmentation of mitochondria (**C1**); (**D**) Effect of hemin (H, grey line), HO-inhibitor ZnIXPP (I, orange line), and hemin plus inhibitor (H+I1, violet line) in the concentrations indicated above on quantification of mitochondrial potential (intensity of background normalized JC-1 fluorescence (emission at 590 nm) was used as a parameter for mitochondrial potential); (**E**) Effect of hemin (H, grey line), HO-inhibitor ZnIXPP (I, orange line), and hemin plus inhibitor (H+I, violet line) in the concentrations indicated above on HO-1 mRNA expression in BRL3A cells determined by qPCR. Data are given as means (±SD) obtained from one representative experiment using *n* = 4 replicates.

Heme toxicity is based on the oxidative modification and consequent damage exerted to membranes and associated proteins when exceeding critical levels. Heme induces HO-1 by directly interacting with the heme-sensitive transcription factors BACH1 and BACH2 [27], resulting in its subsequent degradation by the up-regulated HO activity. HO may provide protection in two ways, either by consumption of heme or by the release of BR, exerting putative anti-oxidative properties, or by both. In the following experiments we examined the effect of HO on hemin-mediated impairment of mitochondrial function. BRL3A cells were treated with 20 µM hemin and mitochondria were visualized using JC-1 (Figure 3), as a measure for membrane potential (Figure 3A1–C1). Membrane potential initially decreased in cells treated with hemin, and this effect was most pronounced when its degradation was inhibited by zinc protoprophyrin IX (ZnIXPP), a competitive inhibitor of HO (Figure 3D). Continuous inhibition of hemin degradation resulted in an increased mitochondrial fragmentation (Figure 3C1). Cells treated with hemin or vehicle alone did not show any effects on their mitochondria (Figure 3A,B).

**Figure 4:**
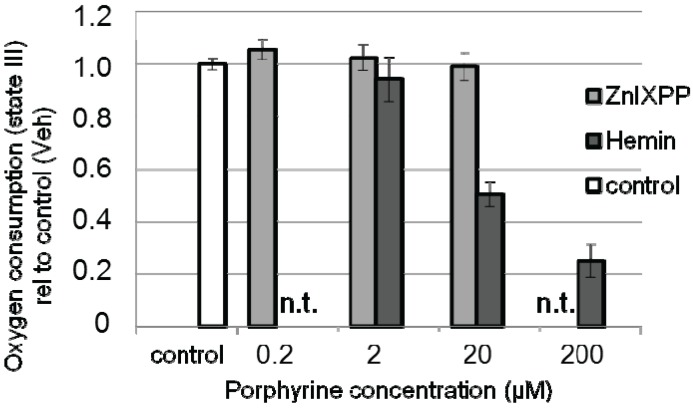
Hemin compromizes respiration of mitochondria. Isolated mitochondria were supplemented with succinate/rotenon to promote respiration of complex II. Transition to state III was induced by adding ADP and oxygen consumption was determined immediately after adding vehicle, hemin in the following concentrations: 2, 20 and 200 µM. In order to exclude effects of the HO inhibitor we also tested ZnIXPP (HO inhibitor) in the following concentrations: 0.2, 2 and 20 µM. Concentrations not tested are indicated (n.t.). State III respiration is indicated relative to the control (vehicle alone, set to 1). Data are given as means (±SD) obtained from one experiment with *n* = 5 replicates.

In order to rule out the possibility that the inhibitor itself may have caused mitochondrial dysfunction, we incubated isolated mitochondria with either hemin or ZnIXPP at various concentrations and examined respiration in terms of oxygen consumption (Figure 4).

We found that isolated liver mitochondria responded with a significant decrease of oxygen consumption at a concentration of 20 µM hemin (Figure 4), which is in line with our previous cell culture experiment. In contrast to hemin, ZnIXPP did not affect mitochondrial respiration, suggesting that iron ions play the principal role in the induction of mitochondrial dysfunction. Thus, our results show that functional HO in parenchymal liver cells protects mitochondria against hemin-mediated respiratory dysfunction. It is possible that the heme degradation products contribute to this effect, although initial levels present are presumably too low, as the levels of hemin are still high. However, longer incubation time may raise BR until reaching effective concentration.

### 3.4. Bilirubin Does not Prevent Hemin-Induced Repression of Respiration in Liver Mitochondria

**Figure 5:**
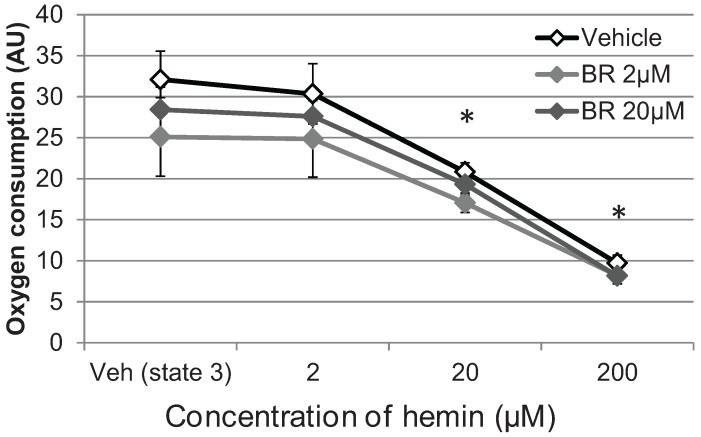
BR was not able to prevent hemin-mediated decrease of mitochondrial respiration. Liver mitochondria were isolated as described in the Materials and Methods section and treated with BR in the indicated concentration or with DMSO (Veh). State 3 respiration of complex II was induced by adding ADP and effects on oxygen consumption were determined after adding hemin in the indicated concentrations. Data are given as means (±SD), obtained from one experiment with *n* = 2 (control)/4 (BR) replicates, indicating significant differences (*) to the control (Veh state 3).

In order to understand whether BR is able to mediate the preservation of mitochondrial function seen in cells with functional HO, we used isolated mitochondria treated with hemin and BR simultaneously (Figure 5). As was shown before (Figure 3 and Figure 4), a nearly immediate dose-dependent decrease of oxygen consumption occurred in mitochondria treated with increasing amounts of hemin. Addition of BR at physiological concentrations tended to further decrease mitochondrial respiration, however, without being significant. Additionally, BR was not able to restore the hemin-mediated depression of respiration (Figure 5). This suggests that in cells treated with hemin, the removal of heme by HO and not the release of BR, mediates the protection of mitochondria.

Interestingly, others showed that BR was able to modulate membrane integrity and redox status [14] of mitochondria, to modulate cytochrome c oxidase activity [28], and to induce apoptotic cell death, which involves mitochondrial pathways [14,29,30], without reporting on changes of respiratory parameters. Thus BR may target other cellular functions that are more sensitive to BR.

### 3.5. Formation of BR and Excretion to the Cell Culture Medium Is Accelerated in Response to hemin, but Decreases Cell Proliferation Rate

We next questioned how much BR is produced by BRL3A cells that are cultured in the presence of hemin, and whether an increased amount of BR extracted from the cell culture medium would reflect the underlying capacity to convert hemin determined *ex vivo* (see Figure 2). The presence of BR in the cell culture medium may represent an additional measure for HO activity *(in-situ* HO activity), provided that BR is not degraded. BR was extracted from medium and extracted into CHCl_3_, and quantified by means of photo spectroscopy using calibration curves.

**Figure 6:**
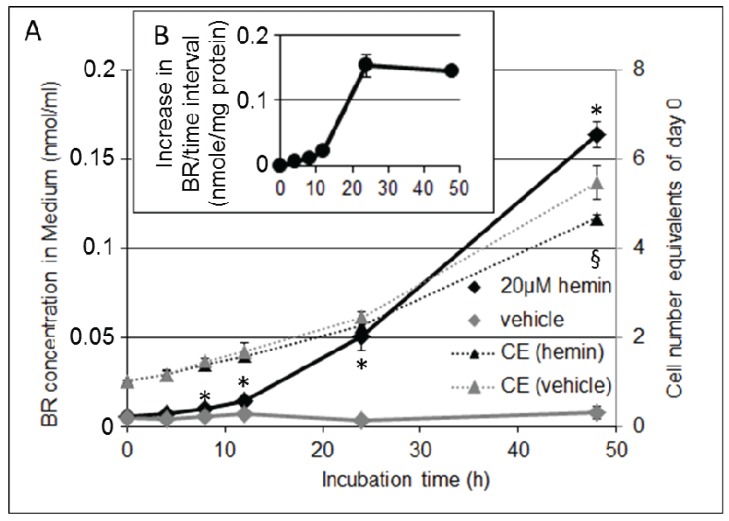
Production of BR by liver cells (BRL3A) as a function of the incubation time. (**A**) Cells were treated with vehicle (DMSO, grey symbols) or hemin (20 µM, black symbols) for the indicated time. BR was extracted from medium (diamonds; solid lines). Underlying cell number equivalents (CE, inset Y-axis) were determined (triangles) using crystal violet assay and expressed relative to the values of day 0 (dotted lines). After 48 h, equivalents of hemin-treated cells were significantly lower compared to the vehicle-control (§); (**B**) Hemin treatment increased the total amount of BR that was newly formed (nmole) per time interval when calculated per mg cell protein. Protein content of cell samples was determined at 48 h using the Bradford method and extrapolated from cell equivalents for each time point. Data are given as means (±SD), obtained from one experiment with *n* = 4 replicates.

Cells treated with 20 µM hemin responded with increasing BR production, compared to control (Figure 6A). We next questioned whether BR production and excretion into the medium would reflect the HO activity determined *ex vivo*. If so, we would expect that an amount of 20 nmol hemin should be converted to 20 nmol BR by 1 mg BRL3A cell protein within 66 h. Considering a fully upregulated HO-1 (approximately within 12 h) we expected 1.6 nmole BR to be produced. However in the time period between 12 h and 24 h we were able to extract only 0.16 nmole BR per mg cell protein (Figure 6B). Although unbound BR may freely diffuse through cell membranes [31], once bound to albumin, a part will be redirected into hepatocytes via vesicular uptake [32]. Furthermore, it was shown that BR may be oxidized by cytochrome P450 2A [33,34], and a part of BR may have been conjugated. Therefore an unknown amount of BR has possibly escaped from quantification. Although the appearance of BR in the cell culture may not properly represent the underlying HO activity *in situ*, the data show that BR formation occurs much slower than expected. With increasing BR concentrations however, the proliferation of the producing cells slowed down, reaching only 80% of the cell number of the control, verified by estimation of the underlying cell number at each time point (Figure 6A, inset Y-axis). Due to the tight interaction of BR it is possible, that the newly formed BR modulates cell function by binding to suitable proteins. Bilirubin was reported to inhibit proliferation in several cell types [35,36,37].

**Figure 7:**
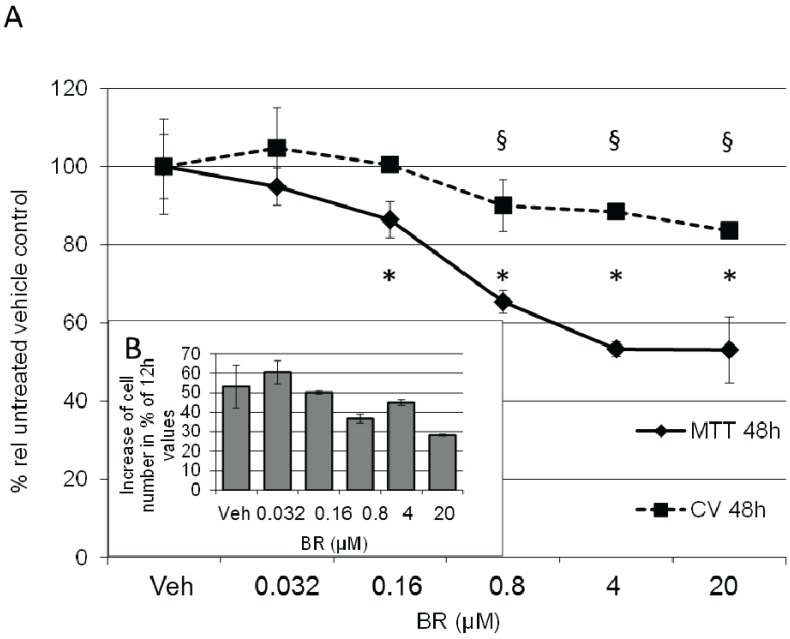
BR results in decreased proliferation and metabolic activity of BRL 3A cells. (**A**) Cells were treated with vehicle (Veh) or BR in the concentrations indicated, and incubated for 48 h. Cell number (squares and dotted line) was determined using crystal violet assay, and expressed in % relative to the vehicle control (CV 48 h). Data are given as means (±SD) obtained from two experiments with *n* = 2 replicates. Metabolic activity of treated cells was determined by MTT assay (diamonds and full line). Data are given as means (±SD) obtained from one experiment with *n* = 4 replicates (MTT 48 h). Significant differences to the control are indicated as (*, §); (**B**) Determination of cell numbers within one experiment at consecutive time points (12 h and 48 h) showed a decreased proliferation rate in the presence of BR. Data are given as % increase relative to the values determined at 12 h.

To elucidate the role of BR in regulating proliferation of BRL3A cells we incubated cells with varying concentrations of unconjugated BR and determined cell number (crystal violet assay; [38,39,40]) and metabolic activity (MTT assay; [41]) at different time points (12 h and 48 h; Figure 7). Considering that an increase in HO would only slowly increase physiologic levels of BR, we used physiologic concentrations of BR, which range between 5 and 32 µM in human serum [42] and about half as much in rodents [43], of which around 4% appears as water-soluble glucuronides [44]. We found that physiologic levels of BR (4–20 µM) decreased the proliferation rate about 20%, but affected the metabolic activity to a much higher degree (50% activity after 48h at the highest BR concentration tested).

### 3.6. BR Increases Expression of Markers for ER Stress and Unfolded Protein Response

Decreased metabolic activity is frequently interpreted as a decrease in mitochondrial energy provision. However, also compromised ER function may lead to decreased cell proliferation rates, especially as a response to ER stressing agents [45].

**Figure 8:**
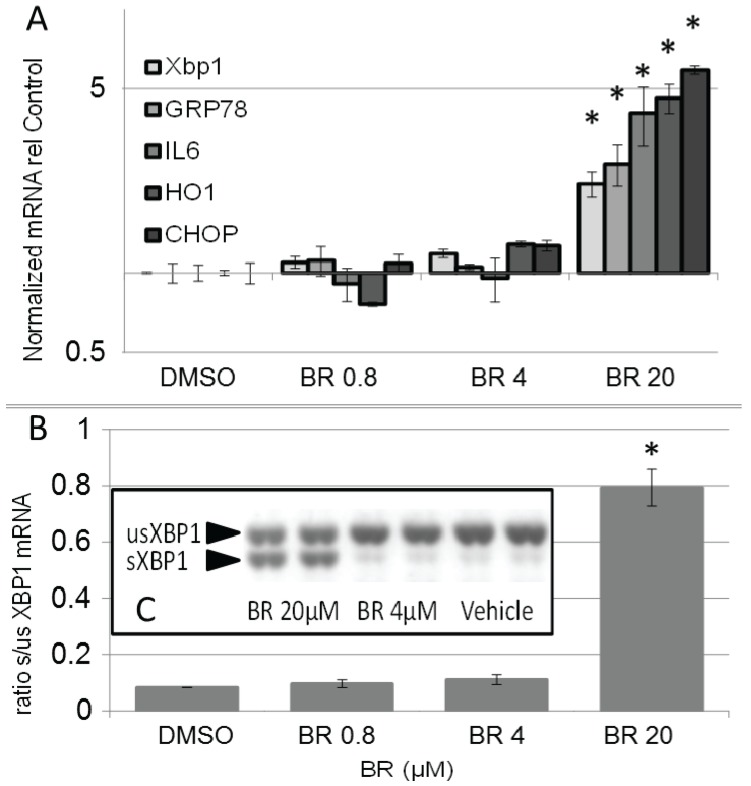
Physiologic range of BR induces ER stress response in BRL 3A cells. (**A**) Cells were treated with vehicle (DMSO) or BR in the concentrations indicated, and incubated for 8 h. RNA was extracted and expression of cell stress markers X-Box binding protein 1 (XBP1), glucose regulated protein 78 (GRP78), interleukin 6 (IL6), HO-1, CRBP homologous protein (CHOP) and the internal reference genes (cyclophilin A, hypoxanthine ribosyltransferase, glycerinaldehyde dehydrogenase) used for basket normalisation was determined by means of real-time PCR. Target mRNA was normalized to the internal references and calculated relative to the vehicle control (DMSO); (**B**) BR induced unconventional XBP1 splicing. PCR products were separated using electrophoresis and visualized by ethidium bromide staining. PCR products consisting of spliced (sXBP1) and unspliced variants (usXBP1) were quantified by means of densitometry using the public domain Scion Image program (http://www.scioncorp.com/), and intensities were expressed as a ratio (spliced to unspliced isoforms). Data are given as means (±SD) obtained from one experiment with *n* = 2 replicates, indicating significant differences (*).

We have found that BR concentrations reduced the metabolic activity of BRL3A indicative for enhanced cell stress. It is known that induction of ER stress decelerates growth rate, involving sXBP1 [46] and promotes apoptosis via CHOP [47,48]. We therefore analyzed the expression of markers for ER stress, X-Box binding protein 1 (XBP1), glucose regulated protein 78 (GRP78) HO-1, CRBP homologous protein (CHOP), and interleukin 6 (IL6) as a marker for an inflammatory response, in BR-treated BRL3A cells. Already after 8 h at concentrations between 4 µM and 20 µM BR elicited an ER stress response (Figure 8), which was accompanied by elevated levels of the XBP1 splice variant, a typical ER-stress marker [49]. Additionally, we determined increased levels of IL6, suggesting onset of an inflammatory response, a pathologic reaction mediated by classical ER stressors [50]. Our data indicate that BR may affect proper function of ER. BR may induce protein mis-folding and aggregation due to its particular chemical properties. We showed that BR, which is newly formed in the HO reaction, tightly binds to proteins. At higher concentrations BR is known to lead to aggregates which are favored at lower pH [51]. Additionally, BR is able to associate with calcium [52], which is high in the ER lumen, and to precipitate with other amphiphilic compounds. This phenomenon is known to occur in the bile leading to the formation of pigmented gallstones containing calcium bilirubinate [53]. In neuronal cells, both mitochondrial and ER function are sensitive to elevated concentrations of BR [54]. In parenchymal hepatocytes, in contrast, BR affects primarily the ER. If the concept remains valid that BR works as a potent anti-oxidant within the cell, an elevated level of BR is supposed to disturb the finely tuned redox equilibrium. However, it is well possible that under conditions of excessive oxidative stress BR would help to reinstall a disturbed equilibrium.

Thus, we cannot answer the question, whether the observed changes would be beneficial under pathologic conditions, or not. It was found, however, that treatment of obese mice with BR over a longer period, relieved signs of metabolic diseases [55]. Interestingly, in this pathologic model, markers for ER stress decreased. Further studies are needed to clarify the significance of BR-mediated interaction with the ER and the induction of a stress response.

## 4. Experimental Section

### 4.1. Chemicals

All reagents were obtained from Sigma-Aldrich (Vienna, Austria) unless otherwise noted. All porphyrins were dissolved in DMSO and used as a 500× stock solution.

### 4.2. Cell Culture

The adherently growing Buffalo rat liver cell line (BRL3A, European Collection of Cell Cultures, Salisbury, UK) was cultivated in Coon’s F-12 medium with 5% FCS (PAA, Linz, Austria). At a confluency of 70%–80% cells were passaged using 0.25% trypsin/EDTA and diluted 1/10 for further culture. Maximal six consecutive passages were used.

### 4.3. Animals

Rats were injected with lipopolysaccharide (LPS) at a dose of 8 mg/kg (i.v.). Adult male Sprague-Dawley rats weighing 280 ± 21 g (Animal Research Laboratories, Himberg, Austria) were divided into two groups: a control group receiving saline i.v., and a group receiving 8 mg lipopolysaccharide/kg i.v. (LPS; *E. coli* 026:B6, Difco, Detroit, MI, USA). At different time points (0, 2, 4, 8, and 12 h (*n* = 3/6)), the animals were killed; liver tissue was taken for analytical examination, aliquoted and stored at −80 °C until analysis. All animals received humane care according to the criteria outlined in the “Guide for the Care and Use of Laboratory Animals” prepared by the National Academy of Sciences and published by the National Institutes of Health (NIH publication 86-23, revised 1985).

### 4.4. Determination of Cell Number by Crystal Violet Assay

BRL3A cells were seeded at a density of 5–10 × 10^4^/mL in 24-wells using Coon’s F-12 medium (5% FCS). The next day medium was exchanged for medium containing hemin (20 µM) or BR (0.032 µM, 0.16 µM, 0.8 µM, 4 µM, and 20 µM) or vehicle (DMSO). Cells were incubated for the given time points (4 h, 8 h, 12 h, 24 h and 48 h or 12 h and 48 h). Thereafter culture medium was removed, cells were washed 3× with PBS and fixed with 4% paraformaldehyde in PBS. After washing 3× with dH_2_O cells were stained using crystal violet staining solution (0.5% in ethanol (10%)) and incubated for 15 min. Thereafter cells were washed three times, plates were dried, and kept in the dark until analysis. In each well 500 µL acetic acid (10%) was added. Stained cells were solubilized by pipetting. One hundred µL of the solution was transferred into a 96-well plate, and extinction at 590 nm was determined using a plate reader.

### 4.5. Determination of Metabolic Activity by MTT Assay

BRL3A cells were prepared as described above in Section 4.4. The next day medium was exchanged for medium containing BR (0.032 µM, 0.16 µM, 0.8 µM, 4 µM, and 20 µM) or vehicle (DMSO). Cells were incubated for 48 h. Six hour prior to the end of the experiment, medium was exchanged for MTT-containing medium (0.5 mg/mL) which was freshly prepared. Following a 6 h incubation period, supernatant was aspirated and formazan crystals were dissolved in sterile DMSO (same volume as the culture medium) by incubating at 37 °C for 30 min. After shaking, 100 µL aliquots were transferred into 96-wells and the absorbance was read at 550 nm using a plate reader.

### 4.6. Cellular Heme Oxygenase Activity by Determination of BR Production in Medium

For determination of BR production, cells were plated in 6-well plates at a density of 5 × 10^4^/mL. The next day medium was supplemented with 0.5% hemin solution (solved in DMSO) to a final concentration of 12.5 µg/mL (corresponding to 20 µM). At each time point (4 h, 8 h, 12 h, 24 h, and 48 h) an aliquot of 200 µL medium was removed and supplemented with 100 µL saturated KCl and 2 mL CHCl_3_. After vortexing (3 × 30 s) and centrifugation (250× *g*) the organic phase was harvested, and bilirubin concentration was determined using photo spectroscopy (U-3000, Hitachi, Tokyo, Japan). The samples were repeatedly (3 times) scanned between 600 and 380 nm using the following settings: slit: 2 nm, 120 nm/min, PMT: autogain, high resolution, and the difference in absorption between 450 and 520 nm determined. Samples were run in triplicates and obtained values were averaged. Calculation of the formed bilirubin was obtained using a standard calibration curve. This standard was generated by adding known amounts of bilirubin to Coon’s F12 medium supplemented with 5% FCS, followed by the subsequent extraction of bilirubin. HO activity was calculated as nmole bilirubin formed per ml per 30 min.

### 4.7. Laser Scanning Microscopy

BRL3A cells were grown in Lab-Tek two-chambered cover glasses (Nalge Nunc, Rochester, NY, USA) with cell culture medium (Coon’s F-12 medium). For confocal microscopic investigations, cells were stained with JC-1 (2 µM MitoProbe, Invitrogen, Carlsbad, CA, USA). After 20 min, cells were treated with Zn(II) protoporphyrin IX (0.1; 1; 10 mg/L; Frontier Scientific, Logan, UT, USA). Subsequently hemin was added at a concentration of 12.5 mg/L. Control cells were treated with DMSO (vehicle control). Thereafter cells were washed with Coon’s F-12 medium. Fluorescence of JC-1 at 590 nm was used as parameter for mitochondrial potential. Imaging was performed with an inverted confocal microscope (LSM 510, Zeiss, Oberkochen, Germany) and 63× oil immersion objective. Image analysis was performed with the histogram toolbar (LSM 510, Zeiss). Regions of interests were marked manually and total fluorescence intensity was defined as mean x area + area x threshold.

### 4.8. Gene Expression

**Figure A1:**
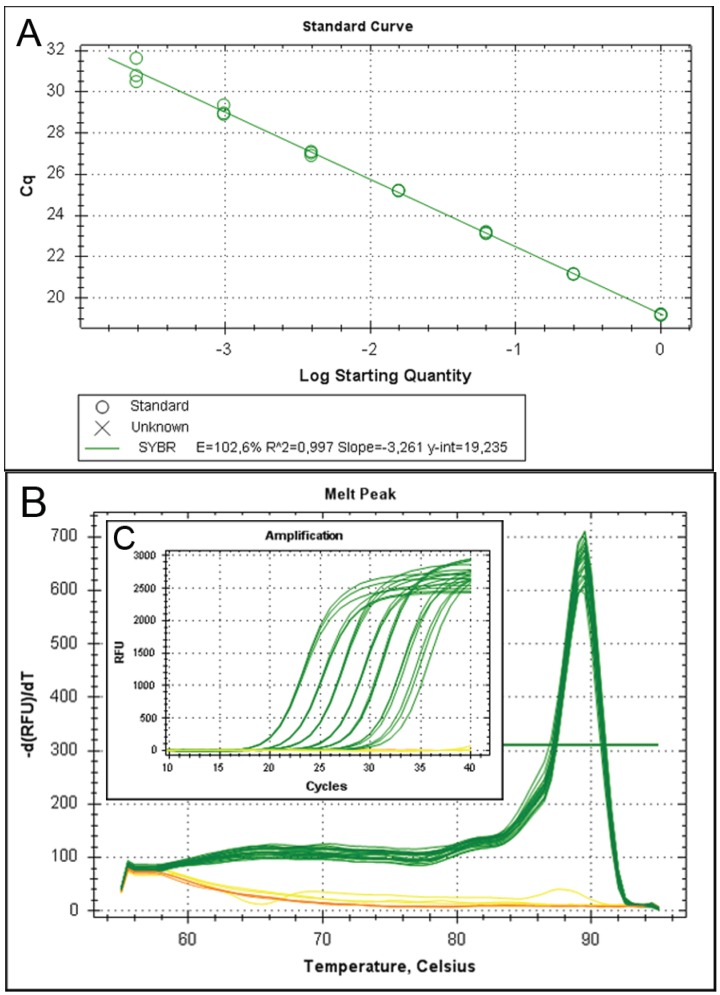
The suitability of the newly designed primers was verified in separate experiments by performing dilution series using the PCR products (Table A2) as well as dilution series of a cDNA pool (**A**). In melt curve (**B**) and amplification plots (**B**) samples are shown in green while controls (no reverse transcription control and no template control) are shown in yellow and orange respectively.

RNA was isolated from BRL3A treated with BR (0.8 µM, 4 µM, 20 µM) for 8 h and processed as described elsewhere [56]. Primer sequences used for amplification are given in Table 1. Primer sequences for XBP-1 were newly designed (and amplification efficiency was verified by dilution series (accessory information is given in the Appendix Figure A1, Table A1 and Table A2). Expression of target genes was measured using a CFX96™ (Bio-Rad, Hercules, CA, USA). Each reaction contained SYBR^®^ green I as reporter (0.5×), iTaq™ polymerase™ (0.625 U/reaction; BioRad), the primers (250 nmol/L each, Invitrogen) with a final concentration of 200 μmol/L dNTP (each) and 3 mmol/L MgCl_2_ in the provided reaction buffer with a final volume of 12 μL. Data were collected in the regression mode and calculated against an internal standard (IS) consisting of pooled cDNA samples of all experiments. We used a modified comparative ΔΔCq method. First the gene specific Cqs were subtracted from the mean Cq of the IS obtained for the same gene giving rise to ΔCq. The values were then subtracted from the normalization factor, which was calculated by averaging the ΔCqs of the internal reference genes (cyclophilin A, hypoxanthinribosyl transferase, glycerinaldehyde dehydrogenase) of the same sample (ΔΔCq). The obtained ΔΔCq values of the replicates were averaged and expressed as 2^−ΔΔCq^ in fold changes relative to the IS.

**Table 1:** Primers used for analysis of gene expression by real-time PCR.

	Accession	Sense Primer	Antisense Primer	Source
XBP-1	NM_001004210.2	gag tcc aag ggg aat gga gt	aca ggg tcc aac ttg tcc ag	Designed for this study
GRP78	S63521	gtt ctg ctt gat gtg tgt cc	ttt ggt cat tgg tga tgg tg	[57]
IL6	NM_012589.1	ccg gag agg aga ctt cac ag	aca gtg cat cat cgc tgt tc	[58]
HO-1	NM_012580.2	cca gcc aca cag cac tac	gcg gtc tta gcc tct tct g	[59]
CHOP	NM_024134.2	ttg ggg gca cct ata tct ca	ctc ctt cag tcg ctg ttt cc	[60]
GAPDH	M17701	cat gcc gcc tgg aga aac ctg cca	tgg gct ggg tgg tcc agg ggt ttc	[61]
HPRT	NM_012583	ctc atg gac tga tta tgg aca gga c	gca ggt cag caa aga act tat agc c	[62]
Cyc	M19533	tat ctg cac tgc caa gac tga gtg	ctt ctt gct ggt ctt gcc att cc	[62]

### 4.9. Determination of Unconventional Splicing of XBP1

For the quantitative determination of the spliced variant of XBP-1 mRNA, 10 µL from the PCR reaction product were separated on a 2% agarose gel and after staining with ethidium bromide visualized by 300 nm UV transillumination. Density of both products, the unspliced and the spliced variant, was quantified via computer assisted densitometric scanning using the public domain Scion Image program (http://www.scioncorp.com/), and the ratio of the spliced to the unspliced variant was determined in each sample.

### 4.10. Heme Oxygenase Activity of Liver Tissue

Liver tissue was homogenized 1:10 (gram tissue/mL buffer) in a buffer containing 300 mM sucrose, 20 mM TRIS and 2 mM EDTA at a pH of 7.4. Approximately 1 mg of protein was added to a reaction mixture containing 500 nmole NADPH in a 100 mM potassium phosphate buffer with 1 mM EDTA (pH: 7.4), supplemented by 20 nmoles of hemin. The mixture was incubated under constant agitation in darkness for 30 min at 37 °C. Afterwards, the reaction was stopped by transferring the samples on ice. After addition of 1/5 volume of saturated KCl, the formed bilirubin was extracted into chloroform (4× the assay volume). Samples were then processed as described in Section 4.6. Samples were run in duplicates and obtained values were averaged and corrected for the absorption measured in corresponding samples incubated at 0 °C. Calculation of the formed bilirubin was obtained using a standard calibration curve. This standard was generated by adding known amounts of bilirubin to a pool of tissue homogenate followed by the subsequent extraction of bilirubin. Protein concentration of liver homogenate was determined using Coomassie Brilliant Blue [63]. HO activity was corrected for the BR lost due to adsorption by proteins using the correction factor described in Section 3.1 and calculated as nmole bilirubin formed per mg protein per 30 min.

### 4.11. Heme Oxygenase Activity of BRL3A Cells

BRL3A cells were seeded at a density of 5–10 × 10^4^/mL in 6-well plates using Coon’s F-12 medium (5% FCS). The next day medium was exchanged for medium containing hemin (20 µM) or vehicle (DMSO). Cells were incubated for 16 h. Medium was discarded, cell layer was washed once with prewarmed PBS and the cells were detached by adding 800 µL of 0.25% trypsin/EDTA. After complete detachment cell suspension of each well was transferred into 5 mL vials containing 4 ml culture medium to stop the trypsin activity. Cells were gently pelleted (400× *g*, RT) (10 min) and supernatant was aspirated. The tube was then placed in liquid nitrogen to snap freeze and stored at −80 °C until being used. For the determination of HO activity, the cell pellet was quickly unfrozen and dissolved in 60 µL buffer containing 300 mM sucrose, 20 mM TRIS and 2 mM EDTA at a pH of 7.4. Approximately 0.3 mg of protein (50 µL) was added to a reaction mixture containing 500 nmole NADPH in a 100 mM potassium phosphate buffer with 1 mM EDTA (assay buffer, pH 7.4), supplemented by 20 nmoles of hemin. The mixture was incubated under constant agitation in darkness for 30 min at 37 °C. Afterwards, the reaction was stopped by transferring the samples on ice. After addition of 1/5 volume of saturated KCl, the formed bilirubin was extracted into chloroform (4× the assay volume). Samples were then processed as described in Section 4.6. Samples were run in duplicates and obtained values were averaged and corrected for the absorption measured in solvent alone. Calculation of the formed bilirubin was obtained using regression analysis of standard calibration curves. These standards were generated by adding known amounts of bilirubin to assay buffer followed by the subsequent extraction of bilirubin. Protein concentration of liver homogenate was determined using Coomassie Brilliant Blue [63]. HO activity was corrected for the BR lost due to adsorption by proteins using the correction factor described in Section 3.1 and calculated as nmole bilirubin formed per mg protein per 30 min.

### 4.12. Preparation of Liver Mitochondria

Sprague-Dawley rats weighing 280 ± 21 g (Animal Research Laboratories, Himberg, Austria) were euthanized by decapitation. The protocol was approved by the City Government of Vienna, Austria, and all experiments were performed under the conditions described in the Guide for the Care and Use of Laboratory Animals of the National Institutes of Health. Immediately after decapitation, liver was extracted and placed in ice-cold sucrose buffer (0.25 M sucrose, 10 mM Tris-HCl, 1 mM EDTA, 0.1% ethanol, pH = 7.4), diced and rinsed with the same buffer to remove remaining blood. After blotting dry with paper, the liver pieces were weighed and the same buffer was added in a ratio of 1:6 liver/buffer (w/v) and homogenized using a Potter-Elvehjem homogenizer. Rat liver mitochondria (RLM) were prepared as described previously [64] and stored at 0 °C for 4–5 h in a buffer containing 0.25 M sucrose, 10 mM TRIS-HCl, 0.5 mM EDTA (pH 7.2), and 0.5 g/L essentially fatty-acid-free bovine serum albumin.

### 4.13. Hepatic Mitochondrial Function

Respiratory parameters of mitochondria isolated from control and LPS-treated rats were determined with a Clark-type oxygen electrode (OROBOROS Ltd, Innsbruck, Austria). Rat liver mitochondria (0.5 mg/mL) were incubated in a buffer consisting of 105 mM·KCl, 20 mM TRIS-HCl, 1 mM diethylenetriaminepentaacetic acid, 5 mM·KH_2_PO_4_, and 1 mg/mL fatty acid-free bovine serum albumin (pH 7.4, 25 °C). Respiration was stimulated by the addition of 10 mM succinate in the presence of rotenone (1 μg/mL; complex II). The transition to state 3 respiration was induced by addition of 200 μM ADP and used as parameter for ATP synthesis.

### 4.14. Data Analysis and Statistics

Data processing and graphics were made using Excel or SPSS 15 (SPSS Inc., Chicago, IL, USA). Data from experiments performed with cells and isolated mitochondria were analyzed by one-way ANOVA followed by LSD post hoc test using SPSS. Data from experiments using liver tissues obtained from animals were subjected to non-parametric analysis using Kruskal-Wallis. Differences to the control were considered significant when *p* < 0.05, and are indicated. The numbers of independent samples (n) are indicated in figure legends.

## 5. Conclusions

We found that HO activity can be determined in each type of sample by the modified photometric extraction assay when the adsorption of BR to protein is accounted for. Additionally this shows that BR that is newly formed by the HO reaction may tightly adsorb to intracellular protein, and thereby modulate the function of sensitive target structures. We found that HO protected mitochondria from hemin-induced toxicity. BR at concentrations that were only slightly higher than the physiological concentrations was capable of inhibiting cell metabolism and proliferation and inducing a stress response at the ER. In BRL3A cells the primary target modulated by BR was the ER, which indicates that HO may modulate ER function *via* newly formed BR.

## Abbreviations

BR: Bilirubin
BV: Biliverdin
Diff. OD: background corrected optical density

## Appendix

**Table A1:** Information about Intron-spanning primers.

Target	Accession number	Start on plus strand	Stop on plus strand	Product-length (bp)	Exon junctions in	Intron size (bp)
XBP-1, transcript variant 1, mRNA (usXBP-1)	NM_001004210.1	435	454	196	Forward Primer Product	~300 ~740
		630	611			
XBP-1, transcript variant 2, mRNA (sXBP-1)	NM_001271731.1	454	473	170	Forward Primer Product	~300 ~740
		623	604			

**Table A2:** Optimized protocol and validation studies using amplificate dilution series

Target	Annealing temp (°C)/time (sec)	Extension temp (°C)/time (sec)	∆Ct (RT+ to RT−)	slope	Correlation-Coefficient (Pearson) R^2^	Verified dynamic range
XBP-1	65/30	72/20	not detected	−3.537	0.997	10^5^
